# Interactions between Inhibitors and 5-Lipoxygenase: Insights from Gaussian Accelerated Molecular Dynamics and Markov State Models

**DOI:** 10.3390/ijms25158295

**Published:** 2024-07-30

**Authors:** Yuyang Liu, Kaiyu Wang, Fuyan Cao, Nan Gao, Wannan Li

**Affiliations:** 1 Edmond H. Fischer Signal Transduction Laboratory, School of Life Sciences, Jilin University, Changchun 130012, China; liuyy1319@mails.jlu.edu.cn (Y.L.); kywang20@mails.jlu.edu.cn (K.W.); 2 Key Laboratory for Molecular Enzymology and Engineering of Ministry of Education, School of Life Sciences, Jilin University, Changchun 130012, China; caofy22@mails.jlu.edu.cn; 3 Key Laboratory of Polyoxometalate and Reticular Material Chemistry of Ministry of Education, Faculty of Chemistry, Northeast Normal University, Changchun 130024, China

**Keywords:** 5-lipoxygenase, Markov state models, GaMD, allosteric inhibition

## Abstract

Inflammation is a protective stress response triggered by external stimuli, with 5-lipoxygenase (5LOX) playing a pivotal role as a potent mediator of the leukotriene (Lts) inflammatory pathway. Nordihydroguaiaretic acid (NDGA) functions as a natural orthosteric inhibitor of 5LOX, while 3-acetyl-11-keto-β-boswellic acid (AKBA) acts as a natural allosteric inhibitor targeting 5LOX. However, the precise mechanisms of inhibition have remained unclear. In this study, Gaussian accelerated molecular dynamics (GaMD) simulation was employed to elucidate the inhibitory mechanisms of NDGA and AKBA on 5LOX. It was found that the orthosteric inhibitor NDGA was tightly bound in the protein’s active pocket, occupying the active site and inhibiting the catalytic activity of the 5LOX enzyme through competitive inhibition. The binding of the allosteric inhibitor AKBA induced significant changes at the distal active site, leading to a conformational shift of residues 168–173 from a loop to an α-helix and significant negative correlated motions between residues 285–290 and 375–400, reducing the distance between these segments. In the simulation, the volume of the active cavity in the stable conformation of the protein was reduced, hindering the substrate’s entry into the active cavity and, thereby, inhibiting protein activity through allosteric effects. Ultimately, Markov state models (MSM) were used to identify and classify the metastable states of proteins, revealing the transition times between different conformational states. In summary, this study provides theoretical insights into the inhibition mechanisms of 5LOX by AKBA and NDGA, offering new perspectives for the development of novel inhibitors specifically targeting 5LOX, with potential implications for anti-inflammatory drug development.

## 1. Introduction

Inflammation is a complex physiological response of the body to tissue damage, infection, or other harmful stimuli [1]. While the inflammatory response is crucial for protecting the body, its dysregulation can lead to chronic inflammatory diseases, such as rheumatoid arthritis [2], asthma, and inflammatory bowel disease [3]. Arachidonic acid (AA) metabolism plays a key role in inflammation [4], with 5-lipoxygenase (5LOX) being the central enzyme in this pathway, catalyzing the conversion of arachidonic acid to leukotrienes [5]. Leukotrienes (e.g., LTB4, LTC4, LTD4, and LTE4) are potent inflammatory mediators involved in leukocyte recruitment and activation, bronchoconstriction, and increased vascular permeability [6]. The aberrant secretion of 5LOX and leukotrienes is closely associated with various inflammatory and allergic diseases, including asthma [7], rheumatoid arthritis [8], and psoriasis [9]. Additionally, the products of the 5LOX pathway are linked to diseases such as cancer [10] and atherosclerosis [11]. Therefore, 5LOX is considered a promising therapeutic target.

**Figure 1:**
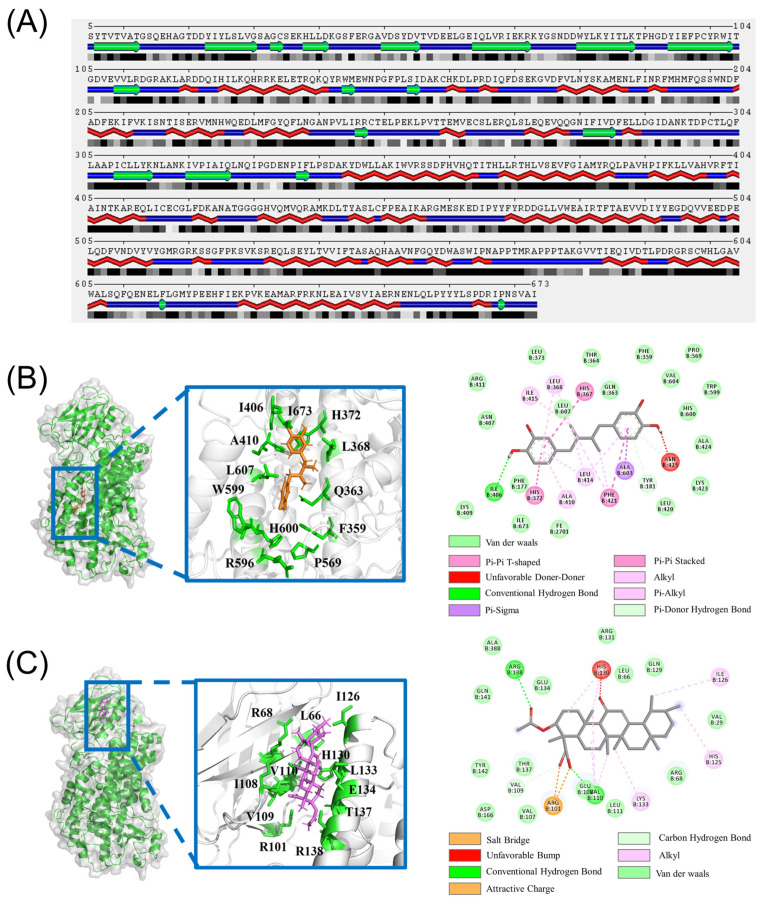
(**A**) Sequence and the secondary structure diagram of 5LOX. (**B**) The active residues around the NDAG inhibitor binding to 5LOX; (**C**) the active residues around the AKBA inhibitor binding to the 5LOX–AKBA complex.

5LOX is composed of 673 amino acids (Figure 1A) and includes two structural domains. The N-terminal regulatory domain, consisting of residues 1–112, is mainly composed of beta sheets, while the C-terminal catalytic domain is significantly larger, spanning residues 125–673. The structural basis of 5LOX function involves non-heme iron in the catalytic site, which is essential for its enzymatic activity [12]. The active site of the enzyme is deeply buried and must undergo conformational changes to access the substrate. Understanding the precise structure of 5LOX and its interactions with inhibitors is crucial for the rational design of effective therapeutic agents.

The 3-acetyl-11-keto-β-boswellic acid (AKBA), a pentacyclic triterpene acid, is a bioactive component of frankincense, known for its well-documented anti-inflammatory properties [13,14]. In 2021, the crystal structures of 5LOX, the orthosteric inhibitor nordihydroguaiaretic acid (NDGA), and the allosteric inhibitor 3-acetyl-11-keto-β-boswellic acid (AKBA) were resolved by Marcia E. Newcomer et al. [15]. The inhibitor NDGA is bound to the active site of 5LOX and interacts with the adjacent residues ILE406, ILE673, HIS372, LEU368, ALA410, LEU607, TRP599, GLN363, PHE359, HIS600, ARG596, and PRO569 (Figure 1B). On the other hand, the inhibitor AKBA is positioned between the membrane-bound domain and the catalytic domain of 5LOX, away from the active center. It interacts with the surrounding residues ARG68, LEU66, ILE126, HIS130, LEU133, GLU134, THR137, ARG138, ARG101, VAL109, ILE108, and VAL110 (Figure 1C).

Although AKBA and NDGA exhibit significant therapeutic potential, the detailed mechanisms of their interactions with 5LOX remain unclear. Due to the presence of high-energy barriers, many biological events occur on the millisecond timescale [16]. Conventional MD simulations often struggle to adequately sample the relevant conformational space of complex biomolecules [17]. Gaussian accelerated molecular dynamics (GaMD) can be used to simulate long-timescale protein dynamics within relatively short computational times. Energy barriers are effectively overcome, significantly enhancing sampling efficiency, which allows for multiple protein conformational states to be explored in a relatively brief period [18,19]. Numerous hundreds-of-nanosecond GaMD simulations have been effectively employed to capture significant conformational changes in various biomolecules. These applications include ligand binding [14,20,21,22,23], protein kinases [24,25], fast-folding proteins [18,26], the CRISPR-Cas9 gene-editing system [27,28], and protein–protein interactions [29,30,31]. The Markov state modeling (MSM) sampling method has emerged as an effective means of studying protein dynamics on millisecond timescales [32,33,34]. The method uses a series of short molecular dynamics (MD) trajectories to sample the conformational space of a protein, identifying a discrete set of inter-transitioning conformational states. Each state is independent and unaffected by previous states. In this way, MSM can combine multiple independently generated MD trajectories to completely characterize the energy landscape of a protein [35,36]. Accelerated molecular dynamics simulations combined with Markov state modeling (MSM) methods show significant potential in the fields of protein engineering and drug discovery [37].

In this study, GaMD was employed to investigate the conformational changes in 5LOX upon binding with the substrate arachidonic acid, the competitive inhibitor NDGA, and the allosteric inhibitor AKBA. Furthermore, the metastable states of proteins were classified using Markov models, revealing the transitions between different conformational states. The molecular mechanisms of 5LOX binding with different ligands were elucidated, providing new insights for the development of anti-inflammatory drugs.

## 2. Results and Discussion

### 2.1. Molecular Docking

**Figure 2:**
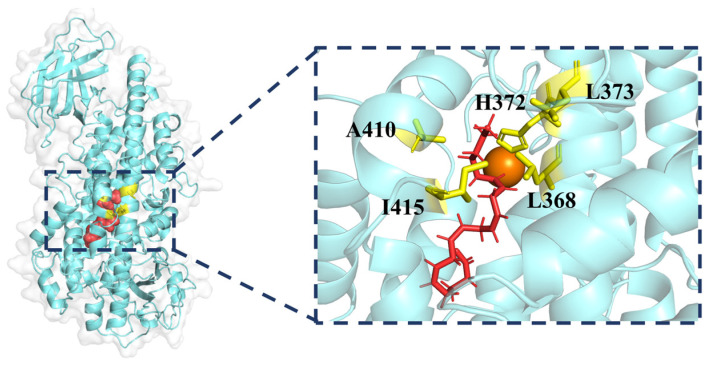
The binding sites of the substrate arachidonic acid (AA) in 5LOX proteins. (The substrate AA is red, FE^2+^ is orange and the active residues are yellow).

The protein–ligand complex of 5LOX and its substrate, arachidonic acid, was subjected to molecular docking to simulate the most probable binding scenario. As Fshown in Figure 2, the substrate arachidonic acid binds tightly to the active pocket of the enzyme and has an alkyl interaction with the proteins LEU368, HIS372, LEU373, ALA410, and ILE415. The substrate binds to the active pocket and has van der Waals interactions with a large number of surrounding residues (Figure S2). The docking results were compared with the S663D Stable-5LOX in complex with arachidonic acid (PDB ID: 3V99), showing that arachidonic acid is docked in within the active cavity close to its position in the crystal structure (Figure S3). After evaluating the structural plausibility, we constructed the corresponding four systems for subsequent molecular dynamics simulations.

### 2.2. Structural Stability of the Four Systems

**Figure 3:**
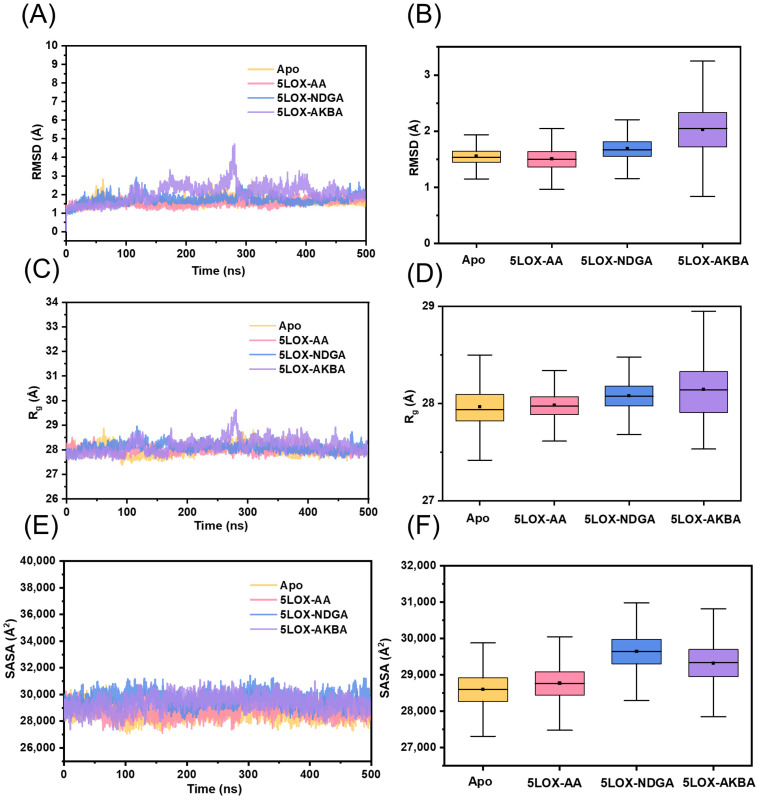
Analysis of structural stability. (**A**) The temporal evolution of the RMSD from their initial structure of the Apo, 5LOX–AA, 5LOX–NDGA, and 5LOX–AKBA systems. (**B**) Distribution of RMSD values in the four systems. (**C**) The temporal evolution of the R_g_ from their initial structure of the Apo, 5LOX–AA, 5LOX–NDGA, and 5LOX–AKBA systems. (**D**) Distribution of R_g_ values in the four systems. (**E**) The temporal evolution of the SASA from their initial structure of the Apo, 5LOX–AA, 5LOX–NDGA, and 5LOX–AKBA systems. (**F**) Distribution of SASA values in the four systems. The median (the horizontal line in the center), the mean (the black dot), and the interquartile range (the upper and lower edges of the box) are shown in the box plot for each set of data.

To evaluate the positional changes in molecular structure and ensure structural stability of each system, we calculated the root-mean-square deviation (RMSD) of the Cα atoms (Figure 3A,B). The average RMSD values for the Apo, 5LOX–AA, and 5LOX–NDGA systems are 1.56 Å, 1.50 Å, and 1.69 Å, respectively (Table 1), indicating minimal changes during the simulation. In contrast, the 5LOX–AKBA system exhibits larger RMSD fluctuations, with an average of 2.03 Å and a variance of 0.49 Å, suggesting significant conformational changes and adjustments in the protein upon binding with AKBA. Over time, the RMSD tends to stabilize, indicating that the protein gradually reaches a new stable state. The stability of ligand binding was assessed by calculating the RMSD values for the regions surrounding the binding sites of NDGA, AKBA, and AA (Figure S4). The RMSD values around AA consistently remained below 0.5 Å, indicating highly stable binding to 5LOX. In contrast, the regions around the binding sites of NDGA and AKBA exhibited greater fluctuations. Furthermore, to reveal changes in protein compactness during the simulation, we analyzed the radius of gyration (R_g_). As shown in Figure 3C,D, the R_g_ value of the 5LOX–NDGA system slightly increases compared to the 5LOX–AA system, indicating that the binding of the competitive inhibitor NDGA induces greater changes in the 5LOX protein. Similarly, the R_g_ value for the 5LOX–AKBA system shows a noticeable increase compared to the Apo system, suggesting conformational changes upon binding with the allosteric inhibitor. In Figure 3A,C, 5LOX–AKBA showed a significant peak at approximately 280 ns. The protein exhibited a significant shift relative to its initial position, causing substantial fluctuations in the RMSD. After 300 ns, the protein returned to its original position. Further analysis revealed the formation of a new helix at residues 280–295 (Figure S5). To further understand the surface properties of the protein during the simulation, we analyzed the solvent-accessible surface area (SASA). As illustrated in Figure 3E,F, the SASA value of the free protein eventually stabilizes, with an average of 28,597.82 Å^2^. The 5LOX–AKBA system shows a significant increase in the SASA compared to the Apo system, with an average value of 29,317.11 Å^2^, indicating that the binding of the allosteric inhibitor enhances the protein’s hydrophilicity. The SASA value for the 5LOX–NDGA system also significantly increases compared to the 5LOX–AA system, suggesting that the binding of the competitive inhibitor NDGA makes the protein conformation more hydrophilic. To ensure the reliability of our results and to prevent chance errors, we performed three independent simulations. We synthesized the results of these three simulations and found that none of them showed large fluctuations at 500 ns, indicating that the system reached equilibrium after this timepoint. We plotted the results of the three simulations as RMSD and Rg plots (Figure S6) and found no significant changes between the different simulation trajectories. Overall, after 500 ns of molecular dynamics simulation, all four systems exhibit good stability and are suitable for further study [38].

### 2.3. Alanine Scanning and the Molecular Mechanics/Poisson–Boltzmann Surface Area (MM–PBSA) 

In this analysis, we conducted mutations using FoldX 5.0 [39] on the residues surrounding the inhibitors NDGA and AKBA by substituting the active groups in their side chains with methyl groups, which have a lesser impact on the protein’s structure. This method was employed to examine the influence of the active residues on the stability of the protein structure. The findings of this investigation are outlined in Table 2 and Table 3. In Table 2, it is evident that the mutations of the residues around NDGA to alanine resulted in minimal changes in energy levels. This observation suggests a strong binding capacity of NDGA to the protein. Conversely, in Table 3, the mutations of the residues LEU66, ARG68, ARG101, HIS125, GLN129, LYS133, and ARG138 to alanine led to an increase in binding energies. This increase implies that these specific sites play a pivotal role in the binding of the inhibitors AKBA and 5LOX. In order to compare the binding ability of substrate AA and orthosteric inhibitor NDGA, the MM–PBSA were calculated as shown in Table 4. The binding energies of AA to 5LOX and NDGA to 5LOX were −28.50 ± 4.11 KJ/mol and −16.18 ± 2.04 KJ/mol, respectively. The substrates AA and 5LOX had smaller binding energies and were more tightly bound.

### 2.4. Flexibility Analysis of the 5LOX

**Figure 4:**
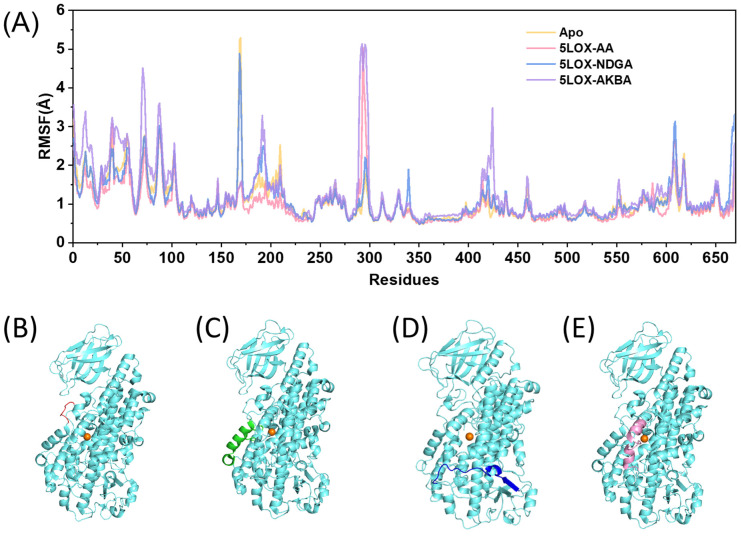
(**A**) RMSF diagrams for the four systems. (**B**) Structure of residues 170–175 in red. (**C**) Structure of residues 176–195 in green. (**D**) Structure of residues 280–300 in blue. (**E**) Structure of residues 406–425 in pink.

To obtain a clearer understanding of protein volatility changes, RMSF (root-mean-square fluctuation) analysis was conducted for the four systems (Figure 4A). At residues 170–175, located near the active cavity of the protein (Figure 4B), the fluctuation of this segment was higher in the empty protein system but significantly reduced after the binding of the inhibitor AKBA. The increased structural rigidity of this protein segment may prevent substrate entry into the active site, thereby inhibiting protein activity. Conversely, upon binding of substrate AA, this amino acid residue exhibited less fluctuation and relative stability around the active site, ensuring efficient catalysis. At residues 176–195, which form the helix α2 in the protein (Figure 4C), the helix α2 of the 5LOX catalytic domain and the neighboring loop were elevated to create a flexible lid that regulates access to the active site. As shown in Figure 4A, the binding of both inhibitors AKBA and NDGA resulted in increased volatility of protein helix α2. This may directly affect substrate entry or product release, thereby impacting the enzyme’s catalytic activity. At residues 280–300 (Figure 4D), where substrate arachidonic acid (AA) binds to the catalytic active site of 5LOX, a local conformational change was observed. The binding of the inhibitor NDGA did not significantly affect the distal structure. However, the fluctuation of the distal loop structure increased upon binding of the inhibitor AKBA, suggesting that AKBA may induce a conformational change in the protein through noncompetitive inhibition. At residues 406–425 (Figure 4E), volatility increased upon AKBA binding. This residue is also located in the helix α2 near the active site. These sites may be critical for reduced protein activity and warrant further investigation.

### 2.5. Conformational Changes during MD Simulations

**Figure 5:**
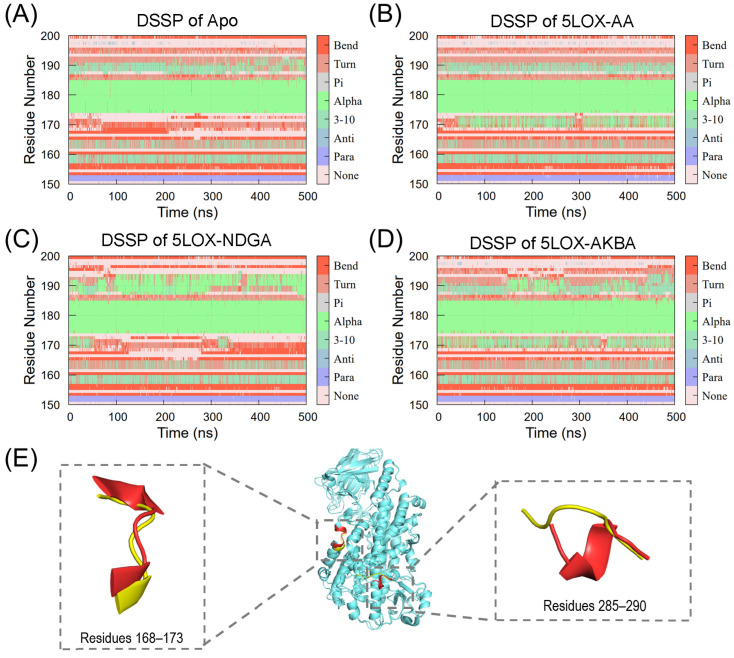
The probability of secondary structure changes in residues 150–200 for (**A**) Apo, (**B**) 5LOX–AA, (**C**) 5LOX–NDGA, and (**D**) 5LOX–AKBA. (**E**) Comparison of the structures of residues 168–173 and residues 285–290 before (yellow) and after (red) the binding of the allosteric inhibitor AKBA.

To compare the conformational changes among the four systems, we analyzed the Dictionary of Secondary Structure of Proteins (DSSP) for two specific regions: residues 150–200 and residues 280–300 of 5LOX. As shown in Figure 5, a significant conformational change occurs in the segment of residues 168–173 in the 5LOX protein. In the Apo system, this segment exists as a loop (Figure 5A), but in the AA-bound state, it gradually transforms into a stable α-helix (Figure 5B). Combined with the RMSF results, the volatility of the 168–173 segment was also significantly reduced (Figure 5D). The structure of the allosteric inhibitor AKBA upon binding is shown in red in Figure 5E. This indicates that, upon substrate binding, this sequence transforms into a more stable α-helix, stabilizing the substrate within the active cavity. The same conformational change was induced upon binding of the inhibitor AKBA. Compared to the null protein, this structure becomes rigid and inhibits the substrate from entering the active cavity, which may be an important reason for the inhibition. Residues 285–290 undergo a similar change (Figure S7). Instead, binding of the inhibitor NDGA occupies the active cavity and causes a change in the conformation of residues 190–195 from a loop to an α-helix (Figure 5C). Upon the binding of the AKBA inhibitor, a more stable α-helix was observed in both residues, which may be a key factor in the inhibition of the protein’s activity. Furthermore, in the AKBA system of replicate simulations, the same structural changes occurred at the same sites (Figure S8), further verifying the statistical significance of our results.

### 2.6. Dynamical Cross-Correlation Matrix Analysis

**Figure 6:**
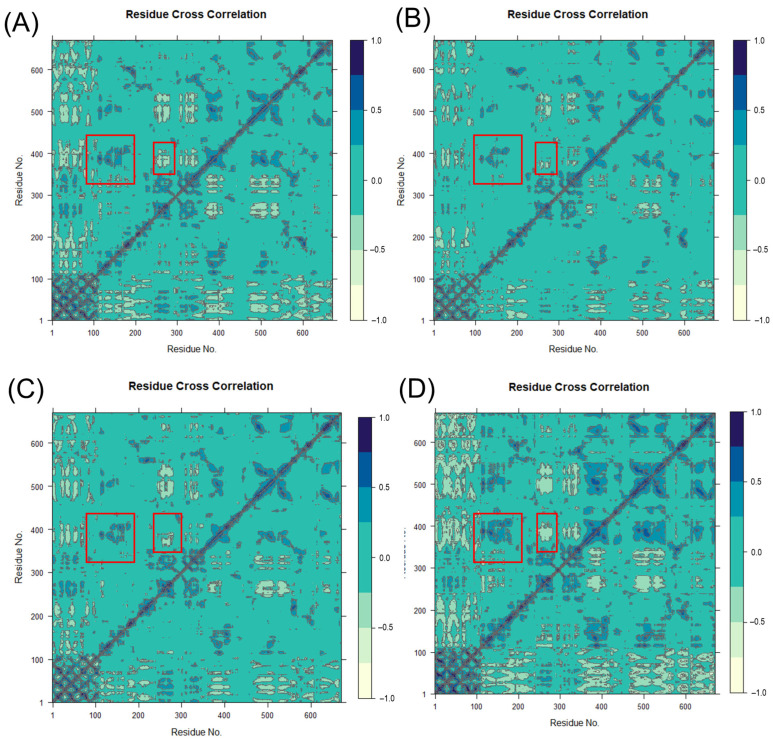
The dynamical cross-correlation matrix diagrams of (**A**) Apo, (**B**) 5LOX–AA, (**C**) 5LOX–NDGA, and (**D**) 5LOX–AKBA systems. (The large red box contains residues 168–173 and 375–425, and the small red box contains 285–290 and 375–400).

The dynamic cross-correlation matrix (DCCM) analysis of all Cα atoms is presented in Figure 6. The relative positions and structure of residues 168–173, 375–425, 375–400, and 285–290 in the 5LOX protein are shown in Figure S9. Significant changes in protein motility were caused by the binding of the allosteric inhibitor AKBA. Residues 168–173 formed a new α-helix upon AKBA binding, and the structure near this residue and the segment of residues 375–425 showed a significant positive correlation of motility (Figure 6D), suggesting that the inhibitor AKBA may indirectly affect 5LOX activity by influencing the conformational stability of the distal region. The covariance matrix of the segments of residues 285–290 and residues 375–400 became lighter in color (Figure 6D), and the negative correlation motions were significantly enhanced, which might have occurred due to relative motions between the structures of these two segments, which in turn affected the binding efficiency of the substrate molecules. This movement further validates the allosteric mechanism of AKBA.

### 2.7. PCA Analysis

**Figure 7:**
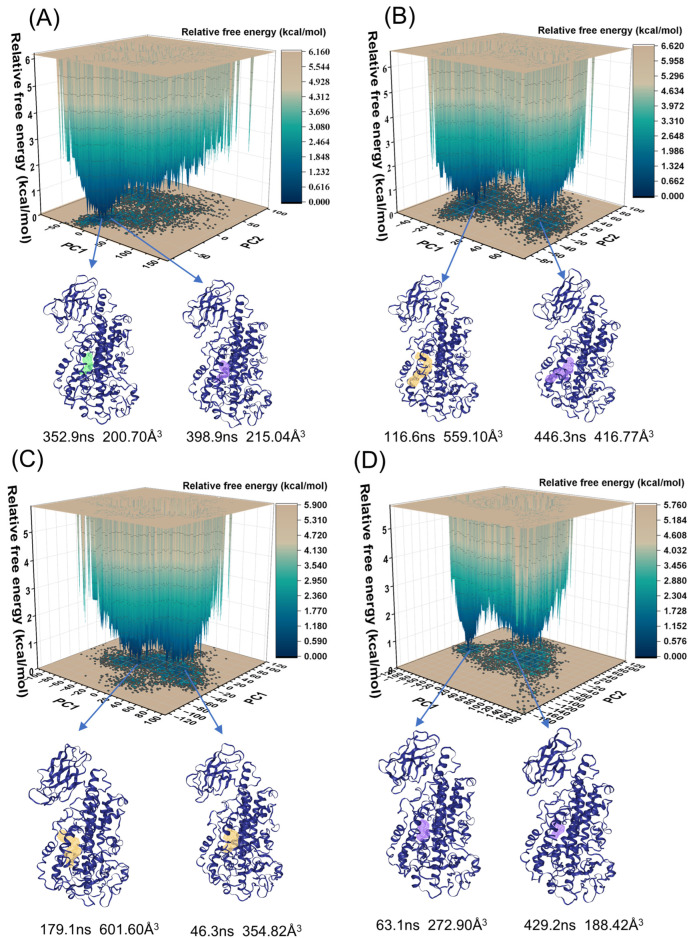
The free-energy landscape for the following four systems: (**A**) Apo, (**B**) 5LOX–AA, (**C**) 5LOX–NDGA, and (**D**) 5LOX–AKBA. The active cavity structures of representative conformations in the low-energy region are indicated by different colors.

The principal component analysis (PCA) of carbon alpha (Cα) atoms was conducted for the four systems to obtain relative free-energy maps (Figure 7). In Figure 7, the protein structure corresponds to the lowest energy state, and the active site cavity is highlighted in different colors. The respective ratios of PC1 and PC2 are listed in Table 5. Table 6 lists the volume and depth of the active cavity at the time corresponding to the lowest energy state of the 5LOX protein during the simulation. The depth of the active cavity is measured by calculating the distance from the deepest point within the binding site to the boundary of the binding site.

In the Apo system, the protein reaches its lowest energy states at 352.9 ns (PC1: −15.88, PC2: −44.05) and 398.9 ns (PC1: 41.57, PC2: −5.81), with corresponding active cavity volumes of 200.70 Å^3^ and 215.04 Å^3^. These observations suggest that the protein cavity volume tends to stabilize over the course of the simulation. Conversely, in the 5LOX–AA system, the protein attains its lowest energy states at 116.6 ns (PC1: −23.60, PC2: −9.94) and 446.3 ns (PC1: 53.60, PC2: −18.47), with active cavity volumes of 559.10 Å^3^ and 416.77 Å^3^, respectively. At 446.3 ns, the protein cavity volume is reduced from the previous measurement, indicating that, over longer time scales, the protein may partially revert to a more compact structure while still maintaining an extended state to some extent. Similarly, in the 5LOX–NDGA system, the protein reaches its lowest energy states at 46.4 ns (PC1: −8.76, PC2: 36.05) and 179.1 ns (PC1: −44.44, PC2: −6.57), with active cavity volumes of 354.82 Å^3^ and 601.60 Å^3^. These results indicate that NDGA binding increases the volume of the protein’s active site cavity, occupying the active site and preventing effective substrate binding. Furthermore, in the 5LOX–AKBA system, the protein shows its lowest energy states at 63.1 ns (PC1: −87.54, PC2: −31.82) and 429.2 ns (PC1: 9.92, PC2: −10.41), with active cavity volumes of 272.90 Å^3^ and 188.42 Å^3^, respectively. Upon AKBA binding, the active cavity volume decreases, suggesting that the protein may have closed certain structural domains, thereby preventing substrate access to the active site. This phenomenon aligns with the conformational change in residues 168–173 (from loop to α-helix) and the enhanced negative correlation motion of residues 285–290. These structural changes increase the rigidity of the active site, further reducing the volume of the active cavity and preventing substrate entry. In conclusion, the binding of AKBA resulted in a more stable and compact structure at the binding site and a significant reduction in the volume of the protein’s active cavity, validating the mechanism by which AKBA inhibits 5LOX through allosteric inhibition.

### 2.8. Markov Model

**Figure 8:**
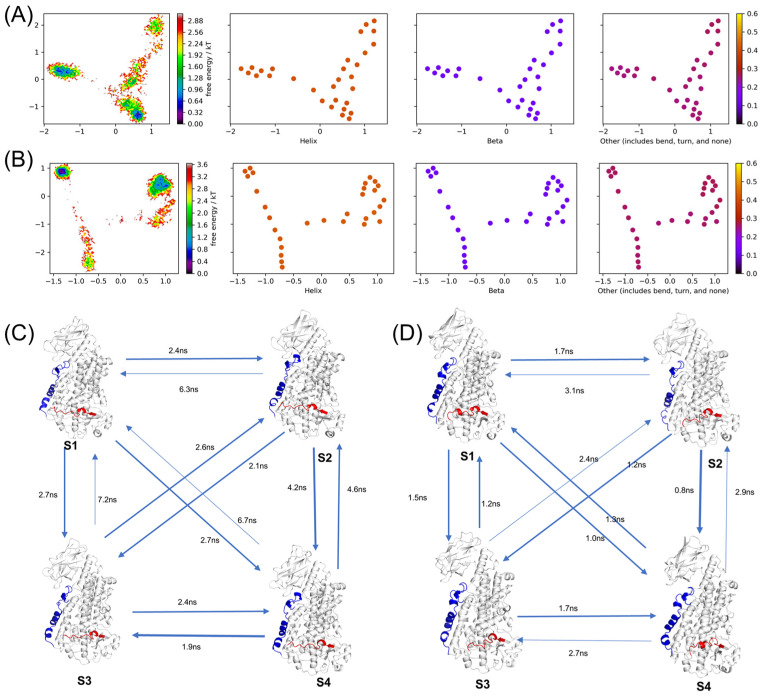
(**A**) Energy landscape colored by secondary structure percentage of 5LOX–NDGA system. (**B**) Energy landscape colored by secondary structure percentage of 5LOX–AKBA system. (**C**) Markov state models and mean first passage time of 5LOX–NDGA. (**D**) Markov state models and mean first passage time of 5LOX–AKBA. (Residues 155–195 are blue and residues 280–295 are red).

To analyze the transitions between different conformational states, the entire Markov model calculation was performed using the Pyemma software package (version 2.5.7) [40]. Trajectories of 5000 frames each, totaling 500 ns, were used for the analysis. The secondary structure information of residues 155–195 and 280–295 were selected for feature extraction, and the results were clustered into 10 classes using the K-Means algorithm. These features were then subjected to time-lagged independent component analysis (TICA). The TICA projection and secondary structure distribution with secondary structure coloring are shown in Figure 8A,B.

After binding with the orthosteric inhibitor NDGA and the allosteric inhibitor AKBA, the protein showed four major low-energy regions in TICA space, corresponding to different stable conformational states. To construct the Markov model, MSMs with different lag times were estimated, and the implied timescales (ITS) were calculated (Figure S10A,B). Lag times of 10 and 5 were chosen for MSM estimation. The validity of the MSM was checked by performing the Chapman–Kolmogorov test (Figure S10C,D). The predicted results matched well with the actual results, indicating that the Markov state model is valid. We identified four substates of the protein and calculated the mean first-passage time (MFPT) for transitions. PCCA (Perron-Cluster Cluster Analysis) [41] was used to color the TICA plots to show the distribution of each substate (Figure S11). The different feature spaces occupied by each state indicate good separation between the states. Finally, the constructed Markov state model was visualized.

In the NDGA-bound protein, the red segment of residues 275–295 remained stable without significant changes, while the blue α2 segment of residues 155–195 showed α-helix formation and disappearance. The transition from state S1 to states S2, S3, and S4 occurred more readily than the reverse, indicating that this segment more easily forms a stable α-helix (Figure 8C). Under the action of the allosteric inhibitor AKBA, the α-helix of residues 280–295 changed. The transition from states S1, S2, and S3 to state S4 took less time, indicating that this transition occurred more easily, and this segment readily formed an α-helix (Figure 8D). State S4 also tended to transition back to state S1, with the segment of residues 280–295 in state S1 forming a more stable α-helix structure. The Markov model analysis results were consistent with the previous PCA analysis results. In conclusion, AKBA binding made it easier for these two regions to transition to a stabilized α-helix, significantly affecting the dynamics and stability of 5LOX. Additional simulations were conducted to further investigate the stability of these substates under equilibrium conditions and their potential transitions. Conventional molecular dynamics simulations of the 5LOX–AKBA systems were performed for 300 ns for each substate structure separately. The results indicate that the RMSD values of each state show minimal fluctuations and stabilize (Figure S12), suggesting that each substate remains relatively stable under equilibrium conditions. Further analysis of the simulation trajectories revealed transitions between different structures within each trajectory. The durations of the different states were recorded during the simulations (Figure S13). These results suggest that transitions between substates are feasible under conventional simulation conditions and that these states can stably exist during the simulations.

## 3. Materials and Methods

### 3.1. Preparation of Four Systems

The 3D structures of 5LOX–AKBA (PDB ID: 6N2W) and 5LOX–NGDA (PDB ID: 6NCF) were downloaded from the RCSB protein Data Bank (www.rcsb.org, accessed on 1 March 2024) [42]. Subsequently, the 5LOX protein was isolated from the protein–ligand complexes using PyMOL software (version number: PyMOL 2.6) [43]. Since the 5LOX protein is a dimer, one of its chains was used in the simulations. Additionally, the structure of the protein residues surrounding NDGA was repaired using homology modeling through the Swiss-Model [44] online platform. Meanwhile, the substrate arachidonic acid (AA) was modeled using Discovery Studio Visualizer (version number: 21.1.0) [45]. Furthermore, Gaussian 16 [46], a widely used software package for quantum chemistry calculations, was employed to optimize the molecular structure at the B3LYP/6-31G* level of theory. The optimized structure obtained from Gaussian 16 [46] then served as the starting point for molecular docking studies. Molecular docking [47], a computational method used to simulate interactions between protein molecules and small ligands, was subsequently performed using AutoDock Vina [48]. In this study, the ligand AA was docked with 5LOX using AutoDock Vina.

In this study, four simulation systems were constructed, as shown in Table 7: Apo, 5LOX–AA, 5LOX–NGDA, and 5LOX–AKBA. Among the ligands, AA was the substrate arachidonic acid, NDGA was the orthosteric inhibitor NDGA, and AKBA was the allosteric inhibitor AKBA. The target protein in this study was 5LOX, which consists of 673 residues and 10,761 atoms.

### 3.2. Molecular Dynamics Simulations

The complexes of the four systems were performed by the PMEMD engine provided with AMBER22 [49]. The forcefield of 5LOX was AMBER ff19SB forcefield [50], and the small molecule field was GAFF2 [51]. The MCPB.py [52] tool was used to process Fe ions and construct the forcefield parameters for the metal ions The PME [53] method was used for treatment of long-range electrostatics. The distance between the solute surface and the box was set to 15 Å, and the box was filled with the OPC water model [54]. Subsequently, sodium ions were randomly added to the simulation box to achieve neutralization of the system, and the steepest descent method was used to minimize the energy. After that, the 100 ps NVT and 100 ps NPT [55] were performed to maintain the system in a stable environment, and the temperature was kept at 310 K by the Langevin thermostat [56] and Langevin pressostat [57] methods. The simulations involved an initial short conventional molecular dynamics simulation of 50 ns to calculate the GaMD acceleration parameters and GaMD equilibration of the added boost potential for 50 ns.

### 3.3. Gaussian Accelerated Molecular Dynamics (GaMD) Simulation

Gaussian accelerated molecular dynamics (GaMD) simulation is a method independent of collective variables (CV), making it widely applicable and beneficial for studying complex biological systems [18]. Its working principle involves adding a harmonic boost potential, which smooths the biomolecular potential energy surface and reduces energy barriers. GaMD significantly accelerates biomolecular simulations by several orders of magnitude [19]. The GaMD boost potential follows a Gaussian distribution, allowing for energy reweighting through a cumulant expansion to the second order (i.e., “Gaussian approximation”). This results in the accurate reconstruction of the biomolecular free-energy landscape. Finally, Gaussian accelerated molecular dynamics (GaMD) simulations with a timestep of 2 fs and a duration of 500 ns were conducted. The simulations were carried out with random initial atomic velocities for each system at the “ dual-boost “ level, where one boost potential was applied to the dihedral energetic term and the other to the total potential energy term. The reference energy was established at the minimum value E = V_max_, while the maximum standard deviation of the boost potential, σ_0_, was designated as 6.0 kcal/mol for both the dihedral and overall potential energy components. To calculate the true free energy, the PyReweighting toolkit [58] was utilized to reweight the trajectory data based on the GaMD simulations. A bin size of 1 Å and a cutoff of 500 frames in each bin were used for the calculation.

### 3.4. MM–PBSA

In this study, the molecular mechanics/Poisson–Boltzmann surface area (MM–PBSA) method was employed to evaluate the binding affinity between ligands and a specific protein. Initially, 5000 frames (500 ns) were selected from the molecular dynamics (MD) simulation, sampling every 10 frames, representing the conformations of both the protein–ligand complex and its individual components for subsequent energy calculations. In the MM/PBSA analysis, the binding free energy (∆G_bind_) is calculated using the following equations:

ΔG_bind_ = ΔH − TΔS (1)

ΔH = ΔE_MM_ + ΔG_sol_ (2)

ΔE_MM_ = ΔE_ele_ + ΔE_vdW_ + ΔE_int_ (3)

ΔG_sol_ = ΔG_pol_ + ΔG_nonpol_ (4)

In these equations, the change in gas-phase molecular mechanical energy is denoted as ΔE_MM_, while ΔG_sol_ represents the variation in solvation free energy. Due to the minimal conformational alterations observed between the receptor and ligand before and after binding, the TΔS term is omitted. ΔE_MM_ comprises three components of molecular mechanics: covalent bonding energy changes (ΔE_int_), electrostatic energy (ΔE_ele_), and van der Waals interactions (ΔE_vdW_). Specifically, ΔE_int_ accounts for changes in bond, angle, and torsion energies. In this study, only the ΔE_ele_ and ΔE_vdW_ components from Equation (3) were analyzed further. The ΔG_sol_ term encompasses the combined effects of polar (ΔG_pol_) and non-polar (ΔG_nonpol_) solvation free energies. The polar solvation free energy (ΔG_pol_) was calculated by solving the linearized Poisson–Boltzmann equation using the PBSA tool within the AMBER 22 software.

### 3.5. Trajectory Analysis

Trajectory analyses, including RMSD, Rg, SASA, RMSF, and distance analyses, were performed using the cpptraj module of Amber22 [59]. Cross-correlation matrix analysis can identify protein regions with significant conformational changes, aiding in the explanation of interactions between the inhibitor and the protein [60]. During molecular dynamics simulations, the covariance matrix is constructed using the first two eigenvectors to describe the correlation of Cα atoms in the main chain. In this study, the dynamics cross-correlation matrix (DCCM) was calculated using R Studio (R version number: 4.3.3) [61].

### 3.6. PCA and Free-Energy Landscape

Principal component analysis (PCA) is an approach of dimensionality reduction, which can help us to identify those motion modes that have the most direct and profound effect on the overall dynamics of the protein [62,63]. The PCA data obtained using the Amber cpptraj program were processed with the ddtpd (version number: ddtpd 1.3) [64] software to obtain the PC1 and PC2 coordinates corresponding to –LnP. These values were then used to plot a free-energy surface map in Origin, which represents the relative magnitude of the free energy for different conformations.

### 3.7. Markov Model

Markov state models (MSMs) play a crucial role in studying the conformational transitions and functional changes in allosteric proteins [65]. They can identify and describe multiple conformational states, construct transition probability matrices to quantify the probabilities and times of transitions between these states, and build free-energy landscapes to illustrate the energy distributions of different conformations. This aids in understanding protein stability, transition mechanisms, and the kinetic pathways of allosteric proteins [66].

#### 3.7.1. TICA Dimensionality Reduction Method

In this study, we used time-lagged independent component analysis (TICA) to reduce the dimensionality of high-dimensional datasets. TICA is a powerful technique that captures slow collective motions by maximizing the time-lagged covariance between data points, thus identifying the most relevant features that vary over time [66,67]. Firstly, the MD trajectory data were processed to extract relevant features from the secondary structure and relative positions of residues 155–195 and 280–295. These features were then applied to the TICA algorithm. By choosing an appropriating lag time, we ensure that the TICA component captures important temporal correlations in the data. Subsequently, the TICA components were used to project the high-dimensional dataset into a low-dimensional space, effectively reducing the data complexity while preserving the most significant dynamic features.

#### 3.7.2. K-Means Clustering Algorithm

The K-Means clustering algorithm is a widely used, unsupervised, machine learning method for partitioning a dataset into K distinct, non-overlapping clusters. K-Means operates by minimizing the variance within each cluster, ensuring that the datapoints in each cluster are as close as possible to the cluster centroid. In the context of Markov models, K-Means clustering is primarily used to cluster the conformational states in molecular simulation trajectories. Each cluster is defined as a microstate in the Markov model, resulting in a discrete trajectory of microstates. The Markov model can then compute the transition probabilities between the microstates, forming a transition probability matrix [68]. The K-Means clustering algorithm was applied in our study to classify the protein conformations obtained from the molecular dynamics simulations. A total of 2–16 different numbers of clusters were subjected to cluster analysis, and the sum of square error (SSE) was recorded for each number of clusters (as shown in Figure S1). The SSE decreases as the number of clusters increases, but the rate of decrease slows down significantly at a cluster number of approximately 10. By analyzing the resulting 10 clusters, different conformational states were identified, and the dynamic behavior of the protein was described.

#### 3.7.3. Determination of Lag Time

The lag time is crucial for constructing Markov state models (MSMs) from molecular dynamics (MD) simulation data. It defines the interval between frames used to calculate transition probabilities, ensuring the model captures the true dynamic behavior of the system. If the lag time is too short, there will not be enough time for transitions between states to decorrelate, causing the MSM to capture only rapid fluctuations rather than true long-term dynamics. Conversely, if the lag time is too long, important intermediate states and transitions may be missed. An appropriate lag time allows for the MSM to exhibit Markovian properties, accurately reproducing system dynamics [65].

To determine the optimal lag time, the Chapman–Kolmogorov (CK) test is used [40]. This test evaluates the consistency of transition probabilities over different lag times. It compares the directly computed transition probabilities over a selected lag time with those obtained by multiplying transition probabilities over shorter times. If the MSM satisfies the CK equation, it indicates that the chosen lag time is appropriate, ensuring the model’s reliability and accuracy.

## 4. Conclusions

In this study, molecular dynamics simulations were utilized to investigate four different systems, Apo, 5LOX–AA, 5LOX–NDGA, and 5LOX–AKBA, examining the protein changes induced by different ligands binding to 5LOX. The results are as follows. The substrate arachidonic acid was tightly bound to the enzyme’s active pocket and interacted with specific residues (such as HIS600, PRO569, ALA603, and ALA424) through alkyl interactions. The orthosteric inhibitor NDGA was tightly bound in the protein’s active pocket, occupying the active site through competitive inhibition, thereby preventing the effective binding of the substrate. The binding of NDGA increased the volume of the protein’s active site cavity, causing structural changes near the active pocket. The binding of the allosteric inhibitor AKBA caused significant changes at the distal active site, resulting in the conformation of residues 168–173 shifting from a loop to an α-helix, and significant negative correlated motions between residues 285–290 and residues 375–400, reducing the distance between these segments. The stable conformation of the protein in the simulation showed a reduced active cavity volume, hindering the substrate’s entry into the active cavity, thus inhibiting protein activity through allosteric effects. Finally, the change paths and transfer times were analyzed using Markov models to verify a more stable change structure. In conclusion, this study reveals the interaction mechanisms and secondary structure changes in 5LOX when bound to various ligands. These findings provide valuable insights for developing potential anti-inflammatory drugs.

## Figures and Tables

**Table 1:** Mean and standard deviation of root-mean-square deviation (RMSD), radius of gyration (R_g_), and solvent-accessible surface area (SASA) in the four systems.

Systems	Average Value			Standard Deviation		
	RMSD (Å)	R_g_ (Å)	SASA (Å^2^)	RMSD (Å)	R_g_ (Å)	SASA (Å^2^)
Apo	1.56	27.97	28,597.82	0.20	0.21	490.91
5LOX–AA	1.50	27.98	28,768.14	0.19	0.14	476.64
5LOX–NDGA	1.69	28.08	29,643.07	0.24	0.16	499.00
5LOX–AKBA	2.03	28.15	29,317.11	0.49	0.29	550.78

**Table 2:** Alanine mutation of residues around NDGA binding.

Mutation	Mutation Energy (kcal/mol)	Effect
PHE177ALA	−0.44	NEUTRAL
TYR181ALA	0.16	NEUTRAL
PHE359ALA	−0.32	NEUTRAL
GLN363ALA	−0.16	NEUTRAL
THR364ALA	−0.08	NEUTRAL
HIS367ALA	0.29	NEUTRAL
LEU368ALA	0.4	NEUTRAL
HIS372ALA	−0.37	NEUTRAL
LEU373ALA	−0.16	NEUTRAL
ILE406ALA	−0.34	NEUTRAL
ASN407ALA	−0.05	NEUTRAL
LYS409ALA	−0.1	NEUTRAL
ALA410ALA	−0.01	NEUTRAL
LEU414ALA	0.83	DESTABILIZING
ILE415ALA	0.25	NEUTRAL
LEU420ALA	−0.04	NEUTRAL
PHE421ALA	−0.2	NEUTRAL
ALA424ALA	0.04	NEUTRAL
ASN425ALA	0.08	NEUTRAL
GLN557ALA	−0.3	NEUTRAL
TRP599ALA	−0.09	NEUTRAL
HIS600ALA	−0.14	NEUTRAL
ALA603ALA	0.04	NEUTRAL
VAL604ALA	−0.11	NEUTRAL
LEU607ALA	0.46	NEUTRAL
ILE673ALA	−0.36	NEUTRAL

**Table 3:** Alanine mutation of residues around AKBA binding.

Mutation	Mutation Energy (kcal/mol)	Effect
VAL29ALA	0.39	NEUTRAL
LEU66ALA	0.77	DESTABILIZING
ARG68ALA	1.27	DESTABILIZING
ARG101ALA	1.04	DESTABILIZING
VAL107ALA	−0.27	NEUTRAL
GLU108ALA	−0.42	NEUTRAL
VAL109ALA	−0.09	NEUTRAL
VAL110ALA	0.05	NEUTRAL
LEU111ALA	0.07	NEUTRAL
HIS125ALA	0.72	DESTABILIZING
ILE126ALA	0.39	NEUTRAL
GLN129ALA	0.74	DESTABILIZING
ARG131ALA	0.28	NEUTRAL
LYS133ALA	0.51	DESTABILIZING
GLU134ALA	−1.06	STABILIZING
THR137ALA	−0.25	NEUTRAL
ARG138ALA	1.02	DESTABILIZING
ASP166ALA	−0.62	STABILIZING

**Table 4:** Molecular mechanics/Poisson–Boltzmann surface area (MM–PBSA) (KJ/mol) of the two systems.

System	5LOX–AA	5LOX–NDGA
ΔE_vdw_	−52.08 ± 3.35	−37.33 ± 0.98
ΔE_ele_	−5.68 ± 4.30	−37.95 ± 5.64
ΔG_solv_	29.26 ± 4.90	59.09 ± 4.08
ΔG_gas_	−57.76 ± 4.54	−75.27 ± 5.18
ΔG_total_	−28.50 ± 4.11	−16.18 ± 2.04

**Table 5:** Principal component (PC) probability of the four systems (%).

Protein	Principle Component (PC)	Probability (%)
Apo	PC1	20.65
	PC2	14.71
5LOX–AA	PC1	13.65
	PC2	10.62
5LOX–NDGA	PC1	13.76
	PC2	12.14
5LOX–AKBA	PC1	27.68
	PC2	13.48

**Table 6:** Active site information for the stabilization structure of the four systems.

System	Time (ns)	Volume (Å^3^)	Depth (Å)
Apo	352.9	200.70	13.46
	398.9	215.04	11.84
5LOX–AA	116.6	559.1	20.25
	446.3	416.77	19.03
5LOX–NDGA	46.4	354.82	13.31
	179.1	601.6	22.47
5LOX–AKBA	63.1	272.90	11.70
	429.2	188.42	12.65

**Table 7:** The four systems of molecular dynamics simulation.

System	Protein	Ligand	SOL
Apo	5LOX	None	10,761
5LOX–AA	5LOX	AA	10,815
5LOX–NGDA	5LOX	NDGA	10,802
5LOX–AKBA	5LOX	AKBA	10,845

## Data Availability

Data is contained within the article and Supplementary Material.

## Supplementary Materials

The following supporting information can be downloaded at: https://www.mdpi.com/article/10.3390/ijms25158295/s1.

## References

[1] Medzhitov R. (2008). Origin and physiological roles of inflammation. Nature.

[2] Hanauer S.B. (2006). Inflammatory bowel disease: Epidemiology, pathogenesis, and therapeutic opportunities. Inflamm. Bowel Dis..

[3] Spiteri A.G., Wishart C.L., Pamphlett R., Locatelli G., King N.J.C. (2022). Microglia and Monocytes in Inflammatory Cns Disease: Integrating Phenotype and Function. Acta Neuropathol..

[4] Harizi H., Corcuff J.B., Gualde N. (2008). Arachidonic-acid-derived eicosanoids: Roles in biology and immunopathology. Trends Mol. Med..

[5] Rådmark O., Werz O., Steinhilber D., Samuelsson B. (2015). 5-Lipoxygenase, a key enzyme for leukotriene biosynthesis in health and disease. Biochim. Biophys. Acta.

[6] Kowal-Bielecka O., Kowal K., Distler O., Gay S. (2007). Mechanisms of Disease: Leukotrienes and lipoxins in scleroderma lung disease--insights and potential therapeutic implications. Nat. Clin. Pract. Rheumatol..

[7] Montuschi P. (2010). Role of Leukotrienes and Leukotriene Modifiers in Asthma. Pharmaceuticals.

[8] Sasaki F., Yokomizo T. (2019). The leukotriene receptors as therapeutic targets of inflammatory diseases. Int. Immunol..

[9] Russo C., Polosa R. (2005). TNF-alpha as a promising therapeutic target in chronic asthma: A lesson from rheumatoid arthritis. Clin. Sci..

[10] Melstrom L.G., Bentrem D.J., Salabat M.R., Kennedy T.J., Ding X.Z., Strouch M., Rao S.M., Witt R.C., Ternent C.A., Talamonti M.S. (2008). Overexpression of 5-lipoxygenase in colon polyps and cancer and the effect of 5-LOX inhibitors in vitro and in a murine model. Clin. Cancer Res..

[11] Bäck M. (2009). Inhibitors of the 5-lipoxygenase pathway in atherosclerosis. Curr. Pharm. Des..

[12] Gilbert N.C., Bartlett S.G., Waight M.T., Neau D.B., Boeglin W.E., Brash A.R., Newcomer M.E. (2011). The structure of human 5-lipoxygenase. Science.

[13] Poeckel D., Werz O. (2006). Boswellic acids: Biological actions and molecular targets. Curr. Med. Chem..

[14] Liao J.M., Wang Y.T. (2019). In silico studies of conformational dynamics of Mu opioid receptor performed using gaussian accelerated molecular dynamics. J. Biomol. Struct. Dyn..

[15] Gilbert N.C., Gerstmeier J., Schexnaydre E.E., Börner F., Garscha U., Neau D.B., Werz O., Newcomer M.E. (2020). Structural and mechanistic insights into 5-lipoxygenase inhibition by natural products. Nat. Chem. Biol..

[16] Hamelberg D., de Oliveira C.A., McCammon J.A. (2007). Sampling of slow diffusive conformational transitions with accelerated molecular dynamics. J. Chem. Phys..

[17] Lazim R., Suh D., Choi S. (2020). Advances in Molecular Dynamics Simulations and Enhanced Sampling Methods for the Study of Protein Systems. Int. J. Mol. Sci..

[18] Miao Y., Feher V.A., McCammon J.A. (2015). Gaussian Accelerated Molecular Dynamics: Unconstrained Enhanced Sampling and Free Energy Calculation. J. Chem. Theory Comput..

[19] Wang J., Arantes P.R., Bhattarai A., Hsu R.V., Pawnikar S., Huang Y.M., Palermo G., Miao Y. (2021). Gaussian accelerated molecular dynamics (GaMD): Principles and applications. Wiley Interdiscip. Rev. Comput. Mol. Sci..

[20] Miao Y., McCammon J.A. (2016). Graded activation and free energy landscapes of a muscarinic G-protein-coupled receptor. Proc. Natl. Acad. Sci. USA.

[21] Wang Y.T., Chan Y.H. (2017). Understanding the molecular basis of agonist/antagonist mechanism of human mu opioid receptor through gaussian accelerated molecular dynamics method. Sci. Rep..

[22] Miao Y., Bhattarai A., Nguyen A.T.N., Christopoulos A., May L.T. (2018). Structural Basis for Binding of Allosteric Drug Leads in the Adenosine A(1) Receptor. Sci. Rep..

[23] Pawnikar S., Miao Y. (2020). Pathway and mechanism of drug binding to chemokine receptors revealed by accelerated molecular simulations. Future Med. Chem..

[24] Moffett A.S., Shukla D. (2020). Structural Consequences of Multisite Phosphorylation in the BAK1 Kinase Domain. Biophys. J..

[25] Koh A., Gibbon M.J., Van der Kamp M.W., Pudney C.R., Gebhard S. (2021). Conformation control of the histidine kinase BceS of Bacillus subtilis by its cognate ABC-transporter facilitates need-based activation of antibiotic resistance. Mol. Microbiol..

[26] Pang Y.T., Miao Y., Wang Y., McCammon J.A. (2017). Gaussian Accelerated Molecular Dynamics in NAMD. J. Chem. Theory Comput..

[27] East K.W., Newton J.C., Morzan U.N., Narkhede Y.B., Acharya A., Skeens E., Jogl G., Batista V.S., Palermo G., Lisi G.P. (2020). Allosteric Motions of the CRISPR-Cas9 HNH Nuclease Probed by NMR and Molecular Dynamics. J. Am. Chem. Soc..

[28] Ricci C.G., Chen J.S., Miao Y., Jinek M., Doudna J.A., McCammon J.A., Palermo G. (2019). Deciphering Off-Target Effects in CRISPR-Cas9 through Accelerated Molecular Dynamics. ACS Cent. Sci..

[29] Sibener L.V., Fernandes R.A., Kolawole E.M., Carbone C.B., Liu F., McAffee D., Birnbaum M.E., Yang X., Su L.F., Yu W. (2018). Isolation of a Structural Mechanism for Uncoupling T Cell Receptor Signaling from Peptide-MHC Binding. Cell.

[30] Park J.B., Kim Y.H., Yoo Y., Kim J., Jun S.H., Cho J.W., El Qaidi S., Walpole S., Monaco S., García-García A.A. (2018). Structural basis for arginine glycosylation of host substrates by bacterial effector proteins. Nat. Commun..

[31] Miao Y., McCammon J.A. (2018). Mechanism of the G-protein mimetic nanobody binding to a muscarinic G-protein-coupled receptor. Proc. Natl. Acad. Sci. USA.

[32] Noé F., Wu H., Prinz J.H., Plattner N. (2013). Projected and hidden Markov models for calculating kinetics and metastable states of complex molecules. J. Chem. Phys..

[33] Husic B.E., Pande V.S. (2018). Markov State Models: From an Art to a Science. J. Am. Chem. Soc..

[34] Olsson S., Noé F. (2017). Mechanistic Models of Chemical Exchange Induced Relaxation in Protein NMR. J. Am. Chem. Soc..

[35] Prinz J.H., Keller B., Noé F. (2011). Probing molecular kinetics with Markov models: Metastable states, transition pathways and spectroscopic observables. Phys. Chem. Chem. Phys..

[36] Olsson S., Wu H., Paul F., Clementi C., Noé F. (2017). Combining experimental and simulation data of molecular processes via augmented Markov models. Proc. Natl. Acad. Sci. USA.

[37] Juárez-Jiménez J., Gupta A.A., Karunanithy G., Mey A., Georgiou C., Ioannidis H., De Simone A., Barlow P.N., Hulme A.N., Walkinshaw M.D. (2020). Dynamic design: Manipulation of millisecond timescale motions on the energy landscape of cyclophilin A. Chem. Sci..

[38] Golla V.K., Prajapati J.D., Joshi M., Kleinekathöfer U. (2020). Exploration of Free Energy Surfaces Across a Membrane Channel Using Metadynamics and Umbrella Sampling. J. Chem. Theory Comput..

[39] Delgado J., Radusky L.G., Cianferoni D., Serrano L. (2019). FoldX 5.0: Working with RNA, small molecules and a new graphical interface. Bioinformatics.

[40] Bowman G.R. (2014). An overview and practical guide to building Markov state models. Adv. Exp. Med. Biol..

[41] Deuflhard P., Huisinga W., Fischer A., Schütte C. (2000). Identification of almost invariant aggregates in reversible nearly uncoupled Markov chains. Linear Algebra Its Appl..

[42] Berman H.M., Westbrook J., Feng Z., Gilliland G., Bhat T.N., Weissig H., Shindyalov I.N., Bourne P.E. (2000). The Protein Data Bank. Nucleic Acids Res..

[43] Schrödinger L.L.C., DeLano W. (2021). Pymol. http://www.pymol.org/pymol.

[44] Waterhouse A., Bertoni M., Bienert S., Studer G., Tauriello G., Gumienny R., Heer F.T., de Beer T.A.P., Rempfer C., Bordoli L. (2018). SWISS-MODEL: Homology modelling of protein structures and complexes. Nucleic Acids Res..

[45] Accelrys Software, Inc. (2021). Discovery Studio Visualizer v21.1.0. https://www.3ds.com/products/biovia/discovery-studio/visualization.

[46] Frisch M.J., Trucks G.W., Schlegel H.B., Scuseria G.E., Robb M.A., Cheeseman J.R., Scalmani G., Barone V., Petersson G.A., Nakatsuji H. (2016). Gaussian 16 Rev. C.01.

[47] Pinzi L., Rastelli G. (2019). Molecular Docking: Shifting Paradigms in Drug Discovery. Int. J. Mol. Sci..

[48] Eberhardt J., Santos-Martins D., Tillack A.F., Forli S. (2021). AutoDock Vina 1.2.0: New Docking Methods, Expanded Force Field, and Python Bindings. J. Chem. Inf. Model..

[49] Case D.A., Cerutti D.S., Cheatham T.E., Darden T.A., Duke R.E., Gohlke H., Goetz A.W., Homeyer N., Izadi S., Janowski P. (2022). AMBER. https://ambermd.org/AmberMD.php.

[50] Tian C., Kasavajhala K., Belfon K.A.A., Raguette L., Huang H., Migues A.N., Bickel J., Wang Y., Pincay J., Wu Q. (2020). ff19SB: Amino-Acid-Specific Protein Backbone Parameters Trained against Quantum Mechanics Energy Surfaces in Solution. J. Chem. Theory Comput..

[51] Wang J., Wang W., Kollman P.A., Case D.A. (2006). Automatic atom type and bond type perception in molecular mechanical calculations. J. Mol. Graph. Model..

[52] Li P., Merz K.M. (2016). MCPB.py: A Python Based Metal Center Parameter Builder. J. Chem. Inf. Model..

[53] Essmann U., Perera L., Berkowitz M.L., Darden T., Lee H., Pedersen L.G. (1995). A smooth particle mesh Ewald method. J. Chem. Phys..

[54] Izadi S., Anandakrishnan R., Onufriev A.V. (2014). Building Water Models: A Different Approach. J. Phys. Chem. Lett..

[55] Bosko J.T., Todd B.D., Sadus R.J. (2005). Molecular simulation of dendrimers and their mixtures under shear: Comparison of isothermal-isobaric (NpT) and isothermal-isochoric (NVT) ensemble systems. J. Chem. Phys..

[56] Bussi G., Parrinello M. (2008). Stochastic thermostats: Comparison of local and global schemes. Comput. Phys. Commun..

[57] Grønbech-Jensen N., Farago O. (2014). Constant pressure and temperature discrete-time Langevin molecular dynamics. J. Chem. Phys..

[58] Miao Y., Sinko W., Pierce L., Bucher D., Walker R.C., McCammon J.A. (2014). Improved Reweighting of Accelerated Molecular Dynamics Simulations for Free Energy Calculation. J. Chem. Theory Comput..

[59] Roe D.R., Cheatham T.E. (2013). PTRAJ and CPPTRAJ: Software for Processing and Analysis of Molecular Dynamics Trajectory Data. J. Chem. Theory Comput..

[60] Hünenberger P.H., Mark A.E., van Gunsteren W.F. (1995). Fluctuation and cross-correlation analysis of protein motions observed in nanosecond molecular dynamics simulations. J. Mol. Biol..

[61] Fischer S., Crow M., Harris B.D., Gillis J. (2021). Scaling up reproducible research for single-cell transcriptomics using MetaNeighbor. Nat. Protoc..

[62] David C.C., Jacobs D.J. (2014). Principal component analysis: A method for determining the essential dynamics of proteins. Methods Mol. Biol..

[63] Chang S., Hu J.P., Lin P.Y., Jiao X., Tian X.H. (2010). Substrate recognition and transport behavior analyses of amino acid antiporter with coarse-grained models. Mol. Biosyst..

[64] Lu T. ddtdp Program. http://sobereva.com/usr/uploads/file/20151115/20151115220021_45515.rar.

[65] Prinz J.H., Wu H., Sarich M., Keller B., Senne M., Held M., Chodera J.D., Schütte C., Noé F. (2011). Markov models of molecular kinetics: Generation and validation. J. Chem. Phys..

[66] Chodera J.D., Noé F. (2014). Markov state models of biomolecular conformational dynamics. Curr. Opin. Struct. Biol..

[67] Rosta E., Hummer G. (2015). Free energies from dynamic weighted histogram analysis using unbiased Markov state model. J. Chem. Theory Comput..

[68] Pérez-Hernández G., Paul F., Giorgino T., De Fabritiis G., Noé F. (2013). Identification of slow molecular order parameters for Markov model construction. J. Chem. Phys..

