# Supplementary material for: Interactions between Inhibitors and 5-Lipoxygenase: Insights from Gaussian Accelerated Molecular Dynamics and Markov State Models
Source: Int J Mol Sci. 2024 Jul 30;25(15):8295. doi: 10.3390/ijms25158295 (PMC11311652; doi:10.3390/ijms25158295)
Supplement: Supplementary file 1 [file ijms-25-08295-s001.zip › ijms-3095465-supplementary.pdf]

Supplementary Materials:

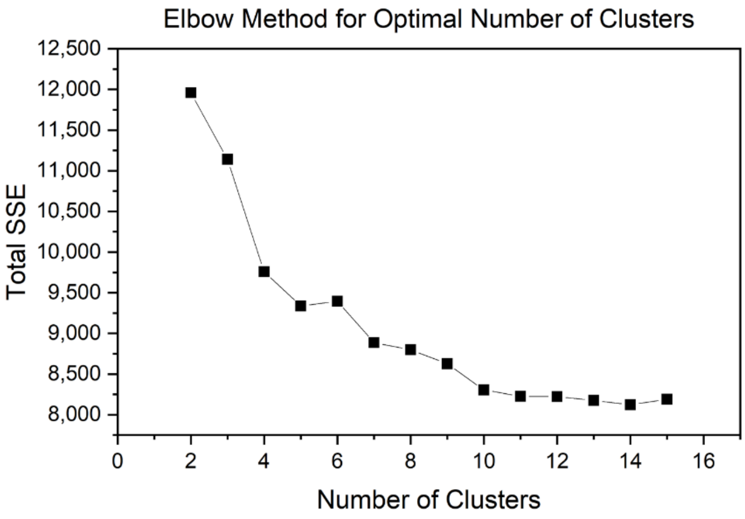

Figure S1. Sum of Square Error for k-means clustering with different number of clusters.

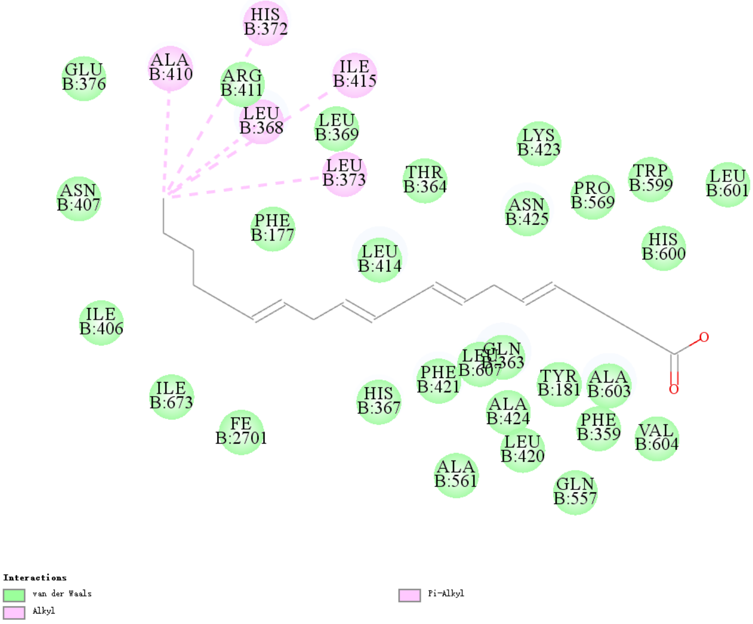

Figure S2. Active residues of substrate AA in 5LOX protein.

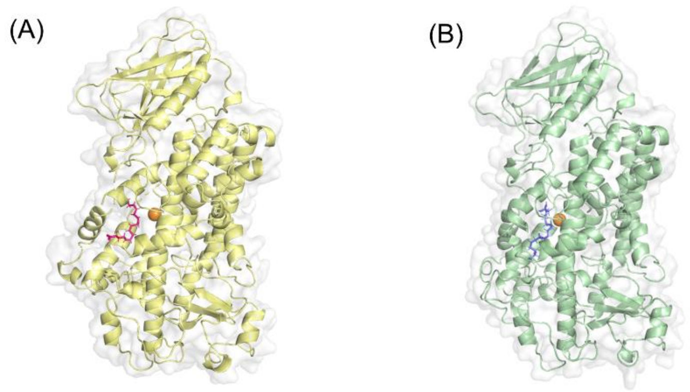

Figure S3. (**A**) S663D Stable-5LOX in complex with Arachidonic Acid (Arachidonic acid is shown in red). (**B**) Results of docking of Arachidonic Acid and 5LOX molecules (Arachidonic acid is shown in blue).

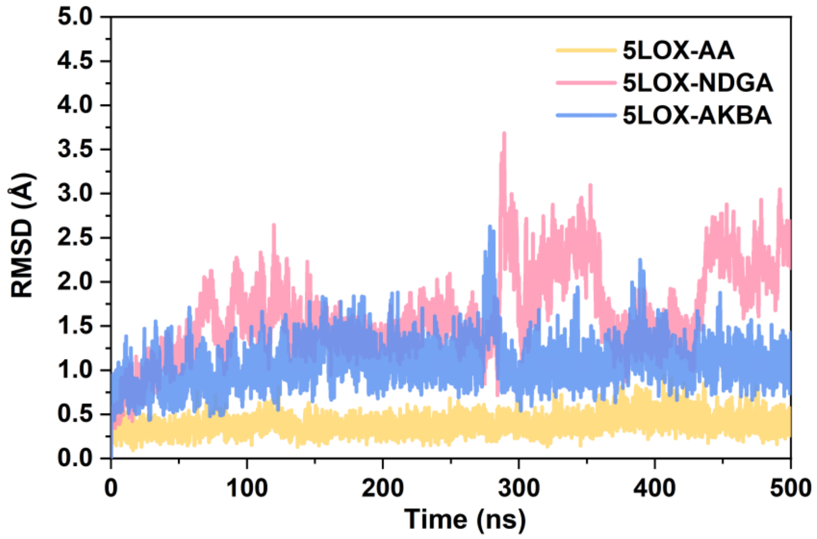

Figure S4. RMSD values for residues around the binding sites of NDGA, AKBA, and AA.

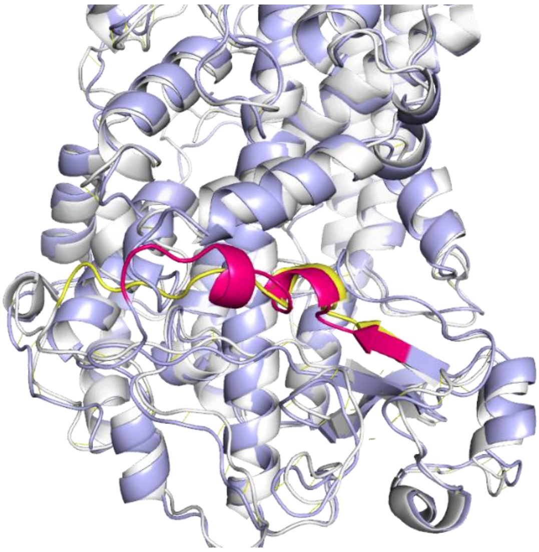

Figure S5. Comparison of the 280ns protein structure (red) with the initial structure of 5LOX (yellow).

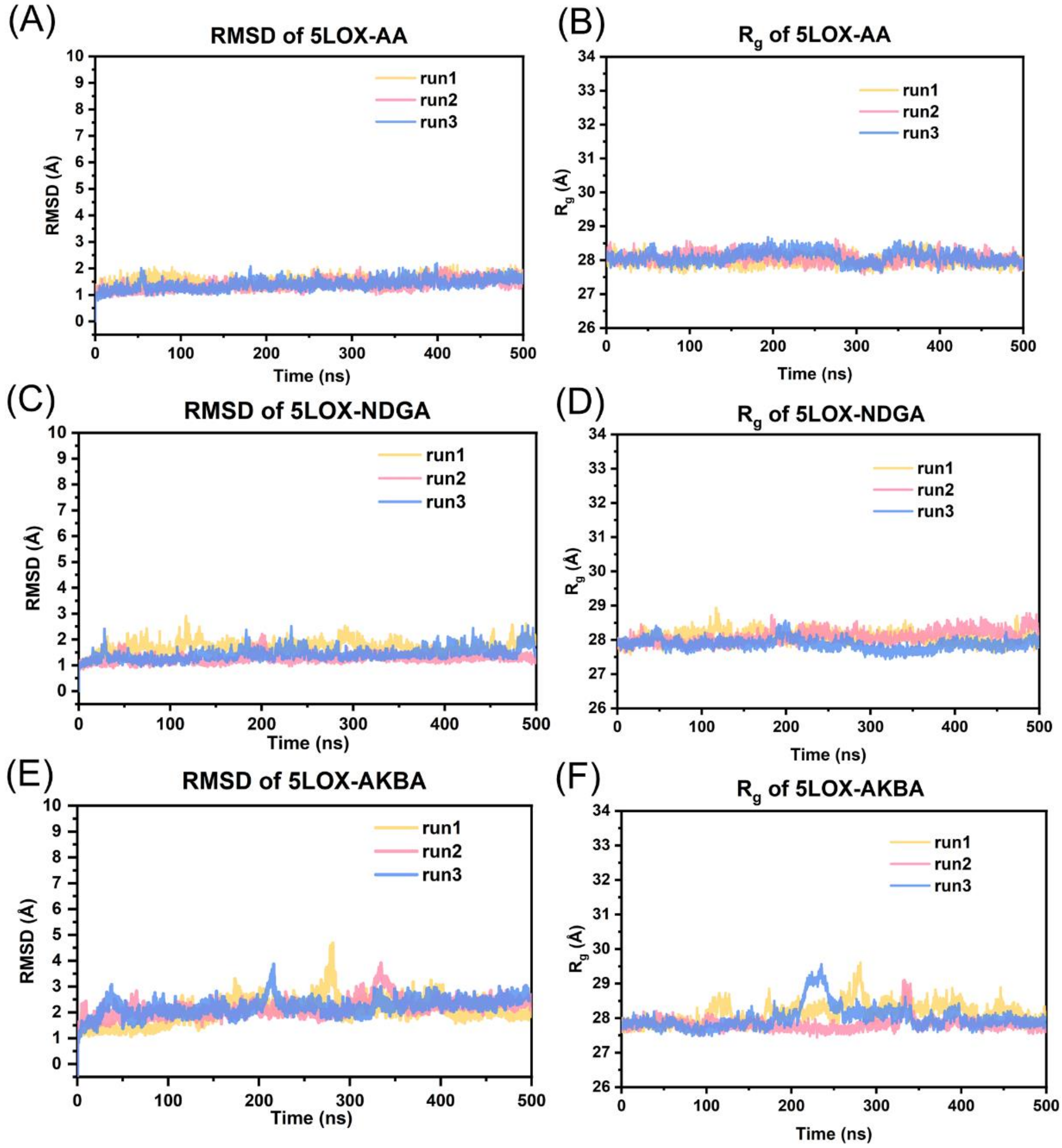

Figure S6. Structural stability analysis with three simulations. (**A**) RMSD of 5LOX-AA. (**B**) R_g of 5LOX-AA. (**C**) RMSD of 5LOX-NDGA. (**D**) R_g of 5LOX-NDGA. (**E**) RMSD of 5LOX-AKBA. (**F**) R_g of 5LOX-AKBA.

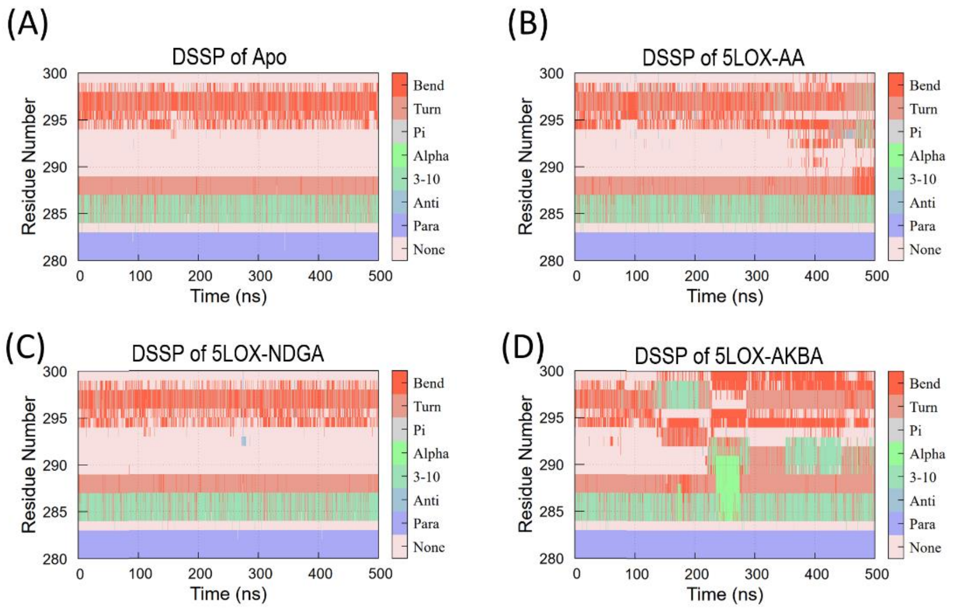

Figure S7. The probability of secondary structure changes in residues 280-300 for (**A**) Apo, (**B**) 5LOX-AA, (**C**) 5LOX-NDGA, and (**D**) 5LOX-AKBA.

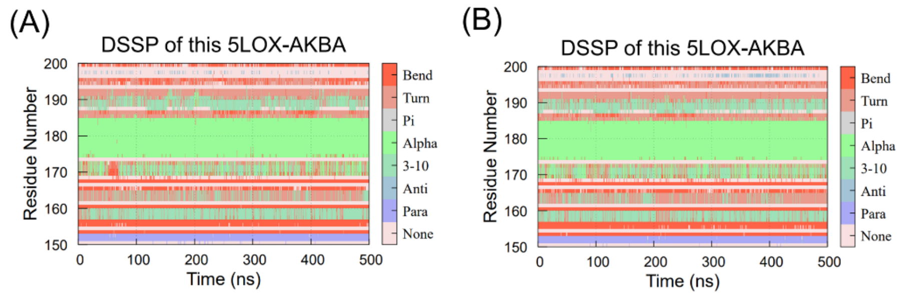

Figure S8. The probability of secondary structure changes in residues 150-200 of 5LOX-AKBA for (**A**) run2 and (**B**) run 3.

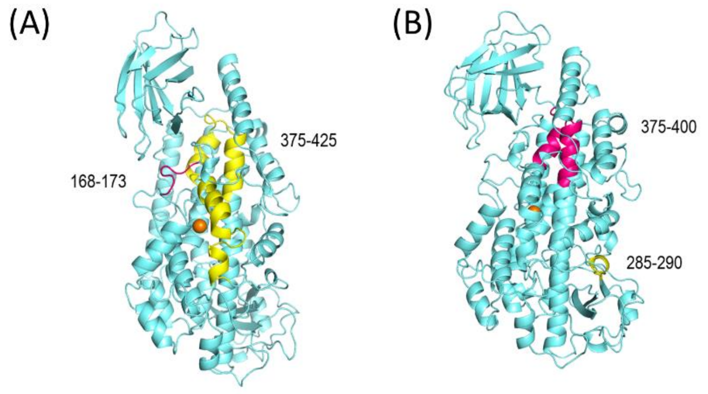

Figure S9. (**A**) Relative positions and structure of residues 168-173 (red) and residues 375-425 (yellow) in 5LOX protein. (**B**) Relative positions and structure of residues 375-400 (red) and residues 285-290 (yellow) in 5LOX protein.

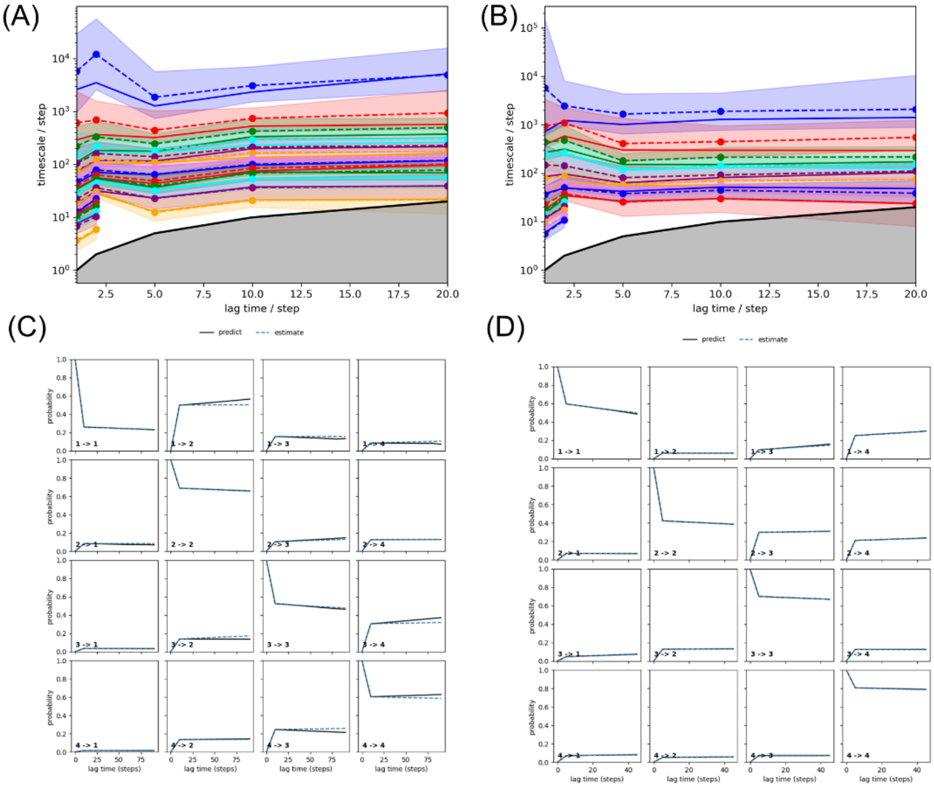

Figure S10. (**A**) Implied time scales of the 5LOX-NDGA system. (**B**) Implied time scales of the 5LOX-AKBA system. (**C**) Chapman-Kolmogorov (CK) test of the 5LOX-NDGA system. (**D**) Chapman-Kolmogorov (CK) test of the 5LOX-AKBA system.

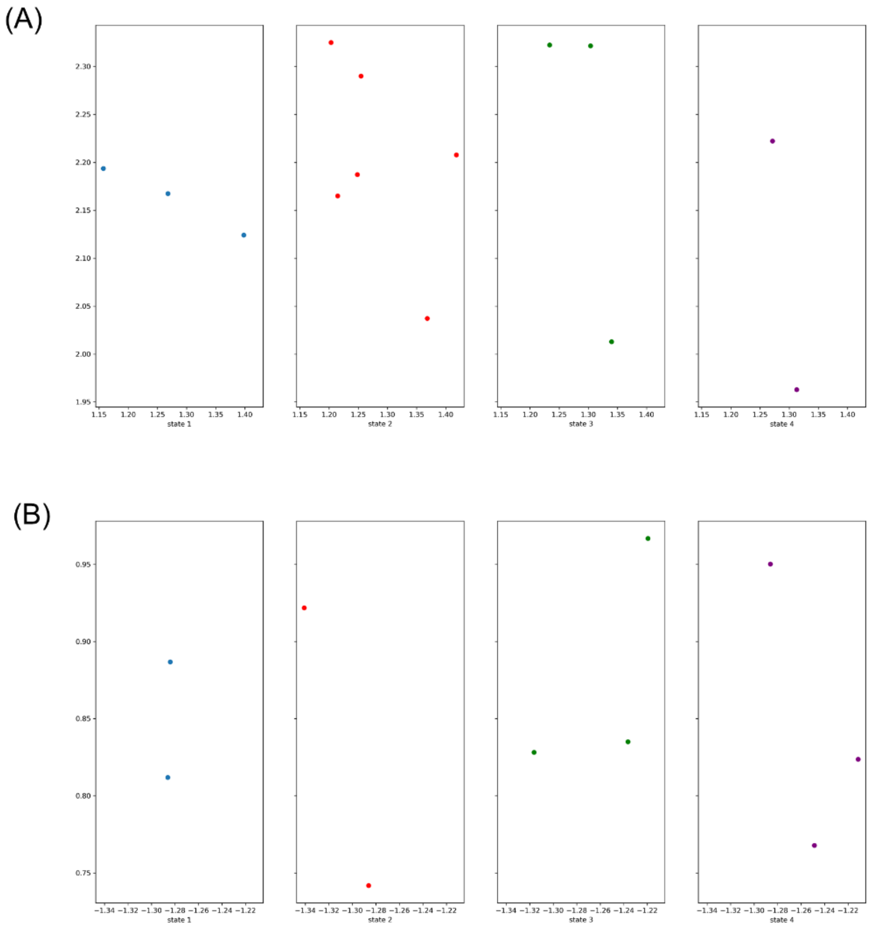

Figure S11. (**A**) Perron-Cluster Cluster Analysis (PCCA) distribution of the 5LOX-NDGA system. (**B**) Perron-Cluster Cluster Analysis (PCCA) distribution of the 5LOX-AKBA system.

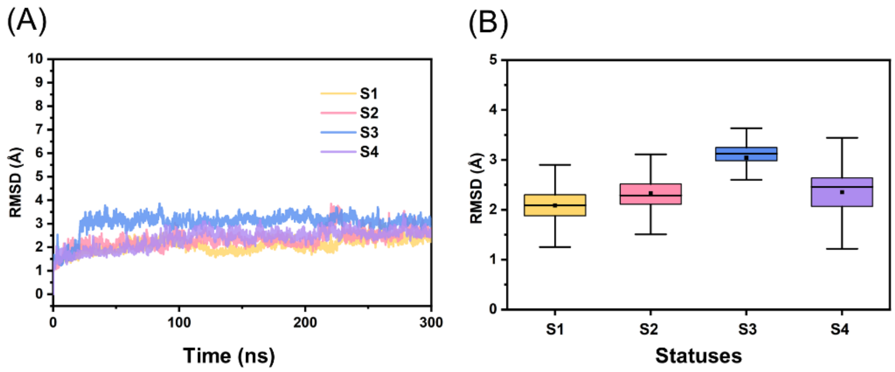

Figure S12. (**A**) RMSD values for metastable states (S1-S4). (**B**) Distribution of RMSD values.

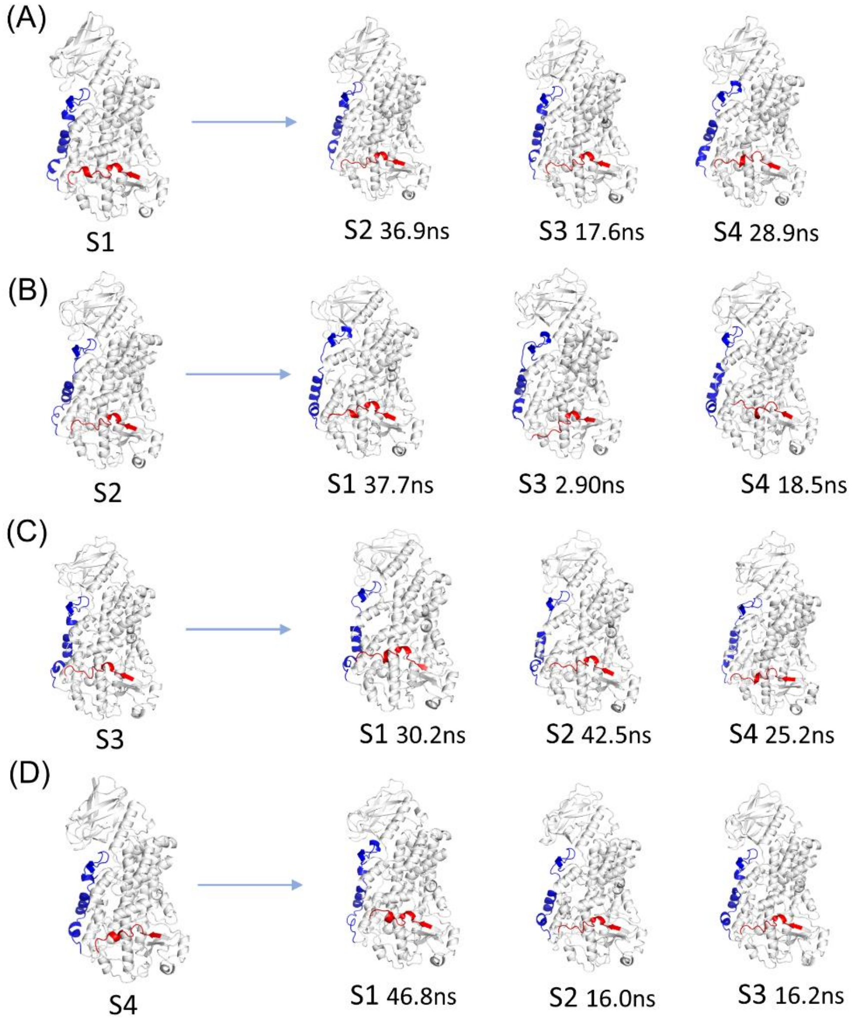

Figure S13. Stable existence times of metastable states S1(**A**), S2(**B**), S3(**C**) and S4(**D**) converted to other states.
